# Developmental and Visual Input-Dependent Regulation of the CB1 Cannabinoid Receptor in the Mouse Visual Cortex

**DOI:** 10.1371/journal.pone.0053082

**Published:** 2013-01-08

**Authors:** Taisuke Yoneda, Katsuro Kameyama, Kazusa Esumi, Yohei Daimyo, Masahiko Watanabe, Yoshio Hata

**Affiliations:** 1 Division of Integrative Bioscience, Institute of Regenerative Medicine and Biofunction, Tottori University Graduate School of Medical Sciences, Yonago, Japan; 2 Division of Neurobiology, School of Life Sciences, Faculty of Medicine, Tottori University, Yonago, Japan; 3 Department of Anatomy, Hokkaido University Graduate School of Medicine, Sapporo, Japan; Tokai University, Japan

## Abstract

The mammalian visual system exhibits significant experience-induced plasticity in the early postnatal period. While physiological studies have revealed the contribution of the CB1 cannabinoid receptor (CB1) to developmental plasticity in the primary visual cortex (V1), it remains unknown whether the expression and localization of CB1 is regulated during development or by visual experience. To explore a possible role of the endocannabinoid system in visual cortical plasticity, we examined the expression of CB1 in the visual cortex of mice. We found intense CB1 immunoreactivity in layers II/III and VI. CB1 mainly localized at vesicular GABA transporter-positive inhibitory nerve terminals. The amount of CB1 protein increased throughout development, and the specific laminar pattern of CB1 appeared at P20 and remained until adulthood. Dark rearing from birth to P30 decreased the amount of CB1 protein in V1 and altered the synaptic localization of CB1 in the deep layer. Dark rearing until P50, however, did not influence the expression of CB1. Brief monocular deprivation for 2 days upregulated the localization of CB1 at inhibitory nerve terminals in the deep layer. Taken together, the expression and the localization of CB1 are developmentally regulated, and both parameters are influenced by visual experience.

## Introduction

Experiences during early postnatal life play an important role in the development of brain function and the refinement of specific neural connections. For example, monocular deprivation (MD) in early postnatal life induces a significant loss of visual cortical responses to the deprived eye in the primary visual cortex (V1) [1], [2]. This so-called ocular dominance plasticity (ODP) exhibits a critical period [2], [3], a postnatal time window in which animals are susceptible to MD, and has been studied as a model of experience-dependent development of neural circuits. Initiation of the critical period requires normal visual experience and the maturation of inhibitory circuit in V1 [4], [5]. Visual experience and postnatal development affect the expression of various molecules that might contribute to ODP in V1 [6]–[9].

Endocannabinoids (eCBs) function as retrograde messengers at synapses that can suppress the release of neurotransmitters and control short- and long-term synaptic plasticity [10]. CB1 cannabinoid receptor (CB1) which localizes at presynaptic terminals is a major cannabinoid receptor in the central nervous system, and 2-arachidonoylglycerol is a major eCB that is synthesized by diacylglycerol lipase-αat postsynaptic sites [11], [12].

In V1 of the rodent, a CB1 antagonist inhibits ODP [13] and CB1 regulates the plasticity of both excitatory synapses [14]–[16] and inhibitory synapses [17], [18] in a layer-specific manner. Although the contribution of CB1 to developmental plasticity is well documented, it remains unclear whether it is regulated by visual experience or postnatal development. In the chick optic tectum, levels of the CB1 protein increase after retinal removal [19]. In the primary somatosensory cortex, the layer distribution of CB1 changes during postnatal development [20]. These reports suggest that CB1 is regulated by activity-dependent mechanisms in an age-dependent manner. To explore a possible role of CB1 in the developmental plasticity of the visual system, we examined the effect of development and visual experience on the protein expression, layer distribution, and synaptic localization of CB1 in mouse V1.

We found intense immunoreactivity of CB1 in layers II/III and VI of V1: this immunoreactivity was more prominently localized at the vesicular GABA transporter (VGAT)-positive inhibitory nerve terminals than at the vesicular glutamate transporter (VGluTs)-positive excitatory nerve terminals. This layer distribution was observed at postnatal day (P) 20 and maintained to P100. The relative amount of CB1 increased from P10 to P100. Dark rearing from birth to P30 decreased the protein expression and altered the synaptic localization of CB1 expression in the deep layer of V1, although the relative amount of CB1 expression was not affected by dark rearing to P50. MD during the critical period affected the synaptic localization of CB1 in the deep layer. These results suggest that the distribution of CB1 matures around the critical period and that visual experience affects the expression and the localization of CB1.

## Materials and Methods

### Animal Treatment

C57BL/6 mice were obtained from Shimizu Laboratory Supplies Co., Ltd. The protocol of the present experiments was approved by the Institutional Animal Care and Use Committee, Tottori University (permission number: 08-Y-42 and 08-Y-71). All surgery was performed under anesthesia with N2O:O2 combined with isoflurane (1.0–4.0%), and all efforts were made to minimize suffering. Normally reared mice were housed under a 12 hr light/12 hr dark cycle. For developmental analysis of CB1, we used mice at postnatal day (P) 10, 20, 30, 40, 50, and 100, with the range of ±1 day. Dark-reared mice were reared in complete darkness from birth to P30 or to P50. Several animals were deprived of vision in one eye by eyelid suture for two days from P27–29 or for seven days from P22–24.

### Antibodies

The primary antibodies that we used in this study are listed in Table 1. We used anti-CB1 antibodies generated from rabbit and goat. The specificities of these antibodies were previously demonstrated by the detection of single protein band at 52 KDa, which was abolished by preadsorption with the antigen protein, in a western blot analysis of a sample of mouse telencephalon [21], [22]. We also confirmed the disappearance of the immunoreactivity of CB1 in V1 by preadsorption with the antigen protein.

**Table 1:** Primary antibodies used in this study.

Primary antibody	Immunogen	Manufacturer, catalog No., species	Concentration/Dilution
CB1	Mouse CB1, C-terminal 31 aa (443–473, NM007726)	Frontier Institute, CB1-Go-Af450, goat polyclonal (Fukudome et al., 2004)	2 µg/ml
CB1	Mouse CB1, C-terminal 31 aa (443–473, NM007726)	Frontier Institute, CB1-Rb-Af380, rabbit polyclonal (Uchigashima et al., 2007),	2 µg/ml for immunohistochemistry, 0.5 µg/ml for western blot
MAP2	Rat brain microtubule associated proteins (MAPs)	SIGMA, M4403, mouse monoclonal	1∶500
Synaptophysin	Vesicular fraction of bovine brain	Millipore, MAB5258, mouse monoclonal	2 µg/ml
VGAT	Mouse VGAT, 31–112 aa (BC052020)	Frontier Institute, VGAT-Rb-Af500, rabbit polyclonal (Fukudome et al., 2004),	2 µg/ml
VGluT1	Mouse VGluT1, C-terminal 531–560 aa (NM20309)	Frontier Institute, VGluT1-Rb-Af500, rabbit polyclonal	2 µg/ml
VGluT2	Mouse VGluT2, C-terminal 550–582 aa (BC038375)	Frontier Institute, VGluT2-Rb-Af720, rabbit polyclonal	2 µg/ml
GAPDH	Glyceraldehyde-3-phosphate dehydrogenase from rabbit muscle	Millipore, MAB374, mouse monoclonal	0.05 µg/ml

### Western Blot Analysis

For western blot analysis, animals were euthanized with an overdose of isoflurane and transcardially perfused with cold 20 mM phosphate-buffered saline (PBS, pH 7.4). Brain tissue was collected immediately and frozen in powdered dry ice. Brains were sliced into 500 µm thickness by a microtome (SM 2000R, Leica Microsystems) and the visual cortical region was quickly dissected. The dissected region was confirmed by observation of residual slices by a microscope (ECLIPSE E800M, Nikon). The tissue was homogenized using a Potter homogenizer with 15 strokes at 3,000 rpm in a homogenizing buffer (0.32 M sucrose, 1 mM EDTA, 1 mM EGTA, and protease inhibitor cocktail (Nacalai Tesque) in 10 mM Tris-HCl (pH 7.4)). The homogenates were centrifuged at 1,000 rpm for 10 min at 4°C and the supernatant was collected. The protein concentration was determined with a Micro BCA Protein Assay Kit (Pierce).

The tissue samples were separated by SDS-PAGE and electroblotted onto PVDF membranes. After blocking by 5% skim milk in 10 mM Tris-buffered saline (pH 7.4) containing 0.1% Tween-20 (T-TBS), the membranes were incubated with T-TBS containing the primary antibodies overnight at 4°C. The membranes were then incubated with HRP-labeled secondary antibody solution (1∶5,000, donkey anti-rabbit antibody; 1∶20,000, sheep anti-mouse antibody, GE Healthcare) for 1 hr. The immunoreaction was visualized with an ECL chemiluminescence detection system (ECL plus or ECL prime, GE Healthcare) and digitalized by a CCD imager (LAS4000, FUJIFILM). Blot densities were quantified using the ImageJ software (Wayne Rasband, NIH, USA).

### Immunohistochemistry

For immunohistochemistry, animals were euthanized with an overdose of isoflurane and transcardially perfused with cold PBS followed by 4% paraformaldehyde in 0.1% PB. Brains were removed from the skull and postfixed in 4% paraformaldehyde and 20% sucrose in PB overnight at 4°C. After postfixation, frozen coronal sections (30 µm in thickness) were prepared with a microtome. All immunohistochemical procedures were performed in a free-floating state.

For immunoperoxidase methods, sections were washed in PBS and incubated in a mixture of 0.5% H2O2, 0.5% Triton X-100 in PBS for 15 min at room temperature to block endogenous peroxidase activity. Then, the sections were incubated in a blocking solution (5% normal goat or rabbit serum (Vector Laboratories), 5% bovine serum albumin (BSA) (SIGMA), 0.5% Triton X-100 in PBS) at room temperature for 4–5 hr. The sections were reacted with the primary antibodies in the blocking solution overnight at 4°C. After washing in PBS, the sections were incubated in the blocking solution for 4–5 hr and then in a secondary antibody solution (1∶200, species-specific biotinylated antibody (Vector Laboratories) in blocking solution) overnight at 4°C. They were then reacted using the conventional ABC-DAB method. All sections were mounted onto MAS-coated slides, dehydrated in an ascending series of ethanol, defatted in xylene, and coverslipped with DPX mountant (SIGMA).

For immunofluorescence, sections were incubated in a blocking solution (5% donkey serum (Jackson ImmunoReseach), 5% BSA, 0.5% Triton X-100 in PBS) for 1–2 hr at room temperature. They were incubated in the blocking solution containing the primary antibodies overnight at 4°C. After washing in PBS, the sections were incubated in a secondary antibody solution (1∶200, Alexa 488-conjugated or Alexa 568-conjugated species specific antibodies (Life Technologies) in the blocking solution) for 2–3 hr at room temperature. After washing, the sections were mounted on MAS-coated slides and coverslipped with Fluoromount/plus (Diagnostic Biosystems).

### Image Analysis

Image analyses were performed using the ImageJ software. Images for horizontal and layer profile analyses of CB1 immunoreactivity in the visual cortex were captured using a cooled CCD camera (VB-7010, Keyence). To measure the horizontal profile of CB1 immunoreactivity, regions of interest (ROIs) were set on layer II/III across cortical areas. Signal intensity was measured in 12 images from 5 animals. To measure the layer profiles of signal intensity for CB1, ROIs (200 µm×800 µm) were set on layer II-VI of the binocular region of V1. CB1 immunoreactivities were measured in 12–20 sites from 3–5 animals in each age group. Layer and region boundaries were defined in neighboring Nissl- or DAPI-stained sections.

For the synaptic localization analysis of CB1, images were acquired with laser confocal microscopy (TCS SP2, Leica Microsystems). Images were obtained using a 63× oil immersion objective lens (NA = 1.4, HCX PL APO, Leica Microsystems) and stored in 8-bit TIFF file format (2,048×2,048 pixels; pixel size, 116.25 nm). The focus was set at a depth of 1–3 µm from the surface of sections. The pinhole size was set at 1.0 Airy unit, and scanning was averaged 8 times. For Alexa 488-labeled samples, the samples were excited by a 488 nm Ar laser, and the beam splitter was set to 505–530 nm. For Alexa 568-labeled samples, the samples were excited by a 543 nm He/Ne laser, and the beam splitter was set to 580–625 nm. The laser power and the gain of the photomultiplier were set to exclude pixels with 0 or 255 intensity in the image. In the figures, the contrast of the images was adjusted for clearer demonstration.

The colocalization of immunofluorescent signals between CB1 and each of synaptophysin, VGAT, VGluT1, and VGluT2 was evaluated by calculating Pearson’s correlation coefficient (CC). Each image was smoothed over 3×3 pixels to remove high frequency noise on the image. We manually set the ROIs (9×9 pixels, approximately 1 µm^2^) at varicosity-like structures and shaft structures in CB1 images. The shaft structure of CB1 was defined as the structure that contains thin fibers with low signal intensity and the varicosity-like structure was defined as the structure that has a large immunopositive area with high signal intensity connected by thin fibers. CC value was calculated as follows:
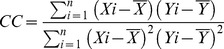
where *X_i_* and *Y_i_* indicate the individual pixel intensities of CB1 and each of synaptophysin, VGAT, VGluT1, VGluT2 in a ROI, respectively. 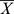 and 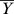 indicate the mean intensity of these components in the ROI. *n* is total number of pixels in the ROI. CC value ranges -1 to 1, and 1 signifies the perfect overlap of two images.

## Results

### Distribution of CB1 in the Visual Cortex

**Figure 1:**
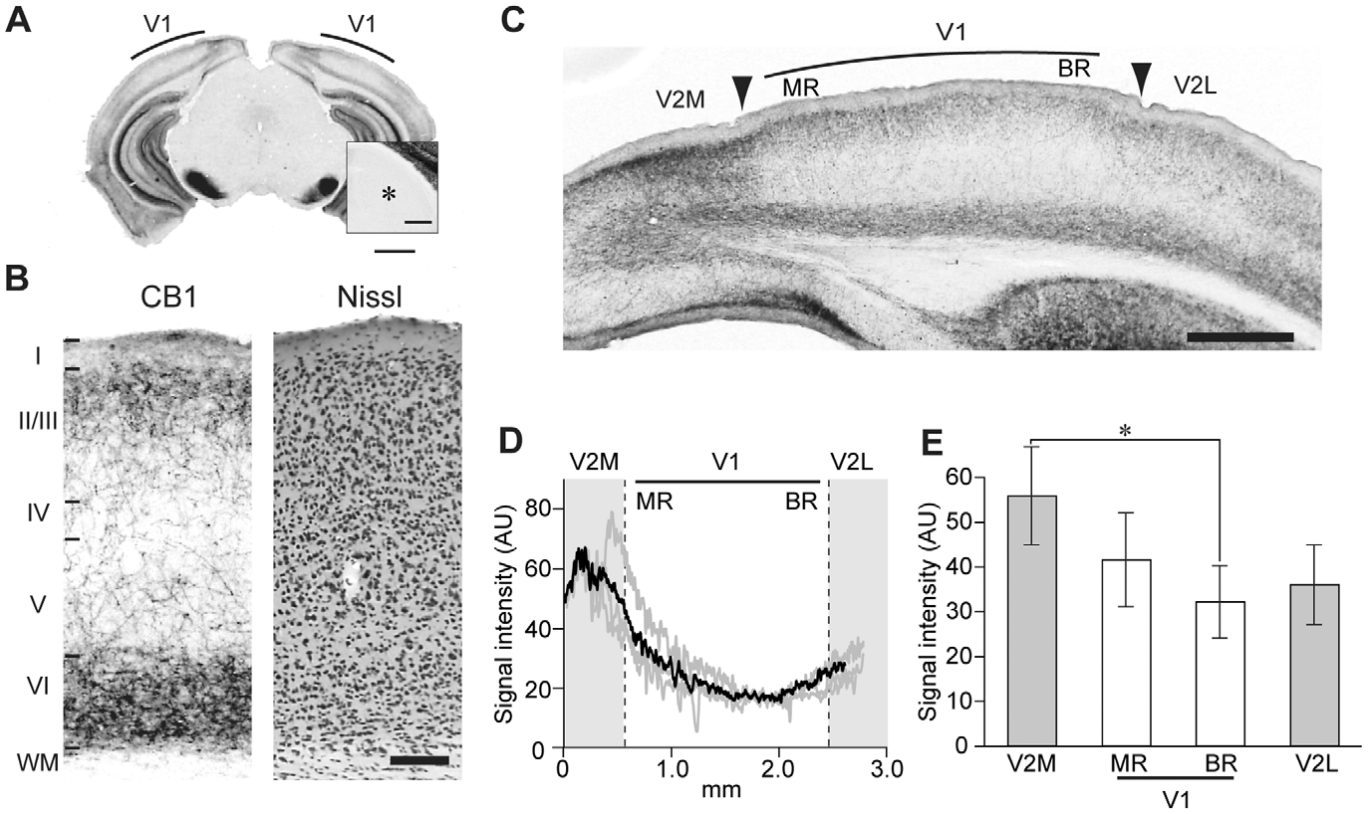
Distribution of CB1 in the visual cortex. (A) Low-magnification image of a coronal section of mouse brain at P30, immunostained for CB1. Inset, magnified view of LGN (*). Scale, 1 mm and 250 µm (inset). (B) Layer distribution of CB1 immunoreactivity in V1 (CB1). Layer boundaries were determined in neighboring Nissl-stained sections (Nissl). Scale, 100 µm. (C) Regional distribution of CB1 immunoreactivity in the visual cortex. Arrowheads indicate the boundaries between V1 and V2, determined in Nissl-stained sections. V2M: secondary visual cortex medial area, V2L: secondary visual cortex lateral area, MR: monocular region, BR: binocular region. Scale, 500 µm. (D) Horizontal profiles of CB1 immunoreactivity across the visual cortex. Signal intensity was measured in layer II/III. Dotted lines indicate region boundaries. The gray lines represent the profiles in individual sections obtained from an animal, and the black line represents the mean of them. AU indicates arbitrary units. (E) Mean signal intensity of CB1 in each visual cortical region. The error bars indicate SEM (n = 5 animals, one-way repeated measured ANOVA, p<0.05, *post hoc* Tukey’s test, *: p<0.05).

We first determined the distribution of CB1 in the visual cortex of P30 mice. Thalami containing the LGN exhibited few immunopositive CB1 signals (Fig. 1A, insert). In V1, the immunopositive CB1 signal was mainly observed as fibrous structures in layers II/III and VI (Fig. 1B). In the visual cortex, an intense CB1 signal, localized in the medial area of the secondary visual cortex (V2M), gradually decreased across cortical regions toward the V1 binocular region (BR) and increased again in the lateral area of the secondary visual cortex (V2L) (Fig. 1C, D). The signal intensity of V2M was significantly higher than that of BR in V1 (Fig. 1E).

### Synaptic Localization of CB1 in V1

**Figure 2:**
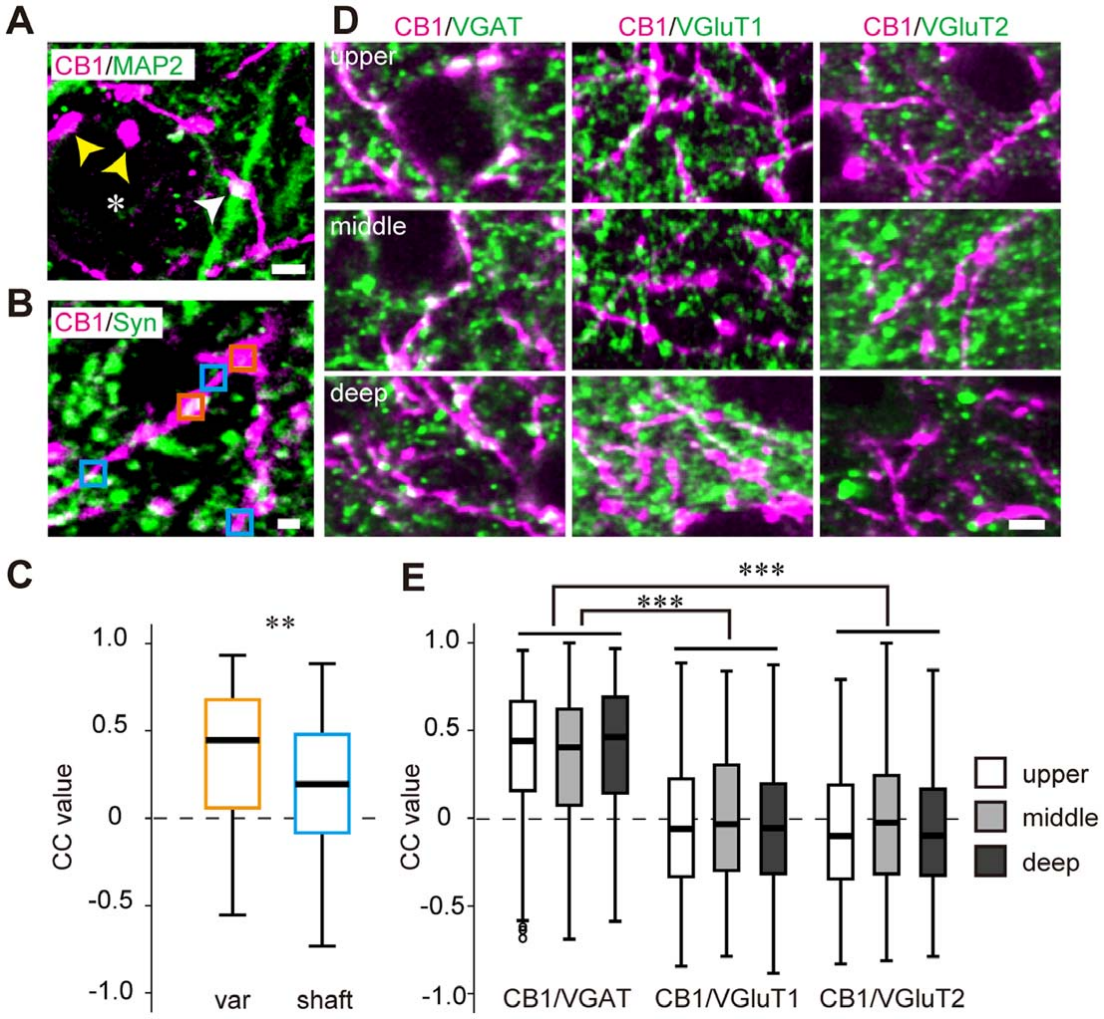
Synaptic localization of CB1 in V1. (A) Double immunofluorescent staining of CB1 (magenta) and MAP2 (green) in the upper layer of V1. CB1-positive varicosities presumably contact MAP2-positive dendrites (white arrowheads) and soma (asterisk, yellow arrowheads). Scale, 3 µm. (B) Double immunofluorescent staining of CB1 (magenta) and synaptophysin (green) in the upper layer of V1. Rectangles indicate the ROIs for the correlation coefficient (CC) analysis set on varicosities (orange) and shafts (blue) of CB1-positive structures. Scale, 1 µm. (C) Box and whisker plots showing the CC values of CB1 and synaptophysin in varicosities (var, n = 154 ROIs) and shafts (shaft, n = 140 ROIs). The horizontal lines show the 25th, 50th, and 75th percentiles, and the whiskers show the max and minimum values. Mann-Whitney U test, **: p<0.01. (D) Double immunofluorescent staining of CB1 (magenta) and VGAT, VGluT1, VGluT2 (green). Representative photographs of the upper layer (top row), middle layer (middle row), and deep layer (bottom row) of V1. Scale, 3 µm. (E) Box and whisker plots showing the CC values of CB1 and VGAT, VGluT1, or VGluT2 in each layer of V1 (n = 6 animals each; in the upper layer, n = 1226 ROIs (CB1/VGAT), 1203 ROIs (CB1/VGluT1), 1212 ROIs (CB1/VGluT2); in the middle layer, n = 492 ROIs (CB1/VGAT), 435 ROIs (CB1/VGluT1), 498 ROIs (CB1/VGluT2); in the deep layer, n = 1556 ROIs (CB1/VGAT), 1712 ROIs (CB1/VGluT1), 1492 ROIs (CB1/VGluT2)). The small circles indicate the outliers of the distribution of the CC values. In the box and whisker plots containing the outliers, the bottom of the whisker shows the value of the 25th percentile-1.5IQR. Statistical comparison among layers was performed by Bonferroni-corrected Mann-Whitney U test (***: p<0.00033).

To elucidate the synaptic localization of CB1, we performed double immunofluorescent staining of CB1 and MAP2 or synaptophysin in the V1 of P30 mice (Fig. 2A, B). An immunopositive CB1 signal was observed in the structures that consist of shafts and varicosities. In the upper layer of V1, CB1-positive varicosities appeared to contact the soma and MAP2-positive dendrites (Fig. 2A). To confirm the presynaptic characteristics of the CB1-positive varicosities, we evaluated the colocalization of CB1 and synaptophysin signals in the CB1 positive varicosities and shafts by calculating CC values (Fig. 2B). The CC value in the varicosities was significantly higher than that in the shafts (Fig. 2C), suggesting the presynaptic nature of CB1-positive varicosities.

CB1 is found in both excitatory and inhibitory nerve terminals [11]. To determine the synaptic localization of CB1 in the V1 of P30 mice, we examined the colocalization of immunopositive signals of CB1 and VGluTs or VGAT. Representative double immunofluorescent staining of CB1 and VGluTs or VGAT is shown in Fig. 2D. We evaluated the colocalization of CB1 and the terminal markers by calculating the CC values in the CB1-positive varicosities. The CC values of CB1 and VGAT were significantly higher than those of CB1 and VGluTs in all cortical layers (Fig. 2E), suggesting that CB1 is mainly localized at the VGAT-positive inhibitory nerve terminals in V1.

### Developmental Changes in CB1 Expression in V1

**Figure 3:**
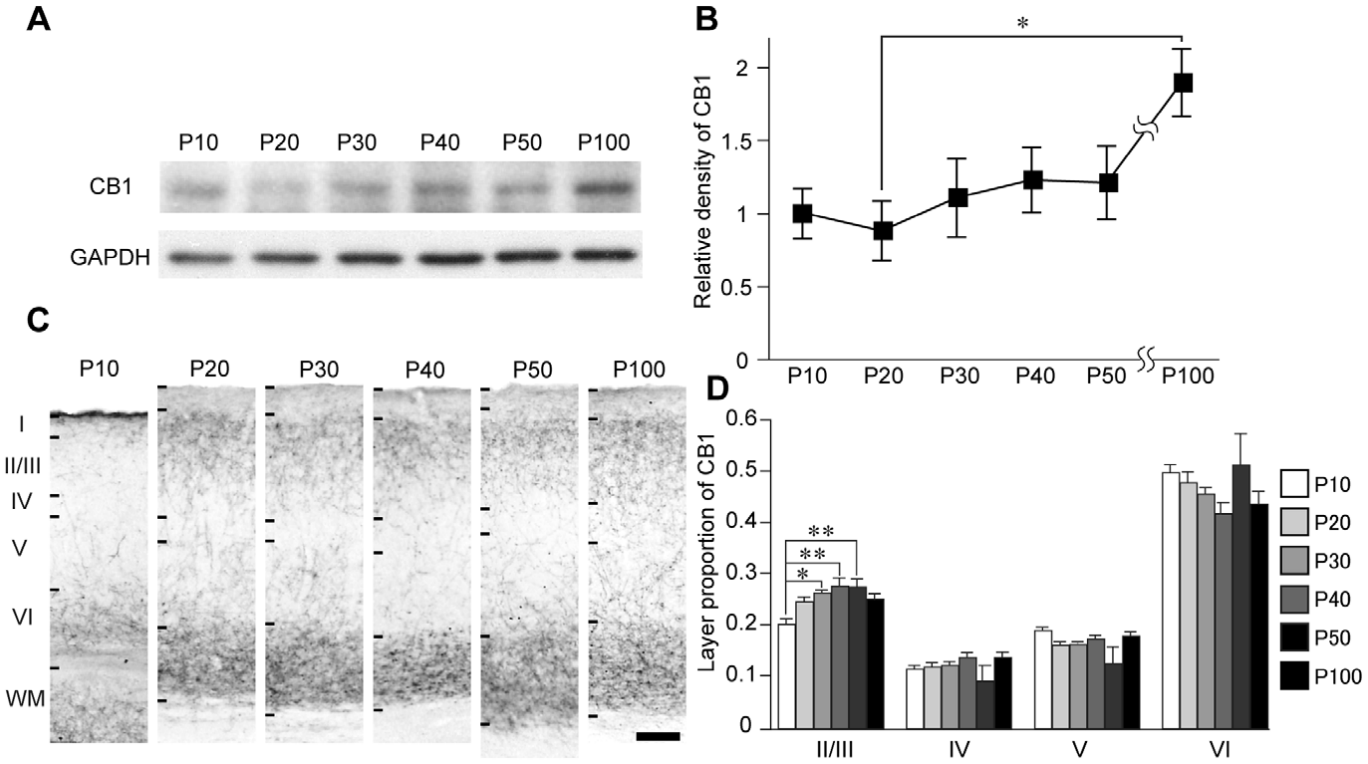
Developmental change of CB1 expression in V1. (A) Representative western blots of CB1 and GAPDH in V1 at different postnatal ages. (B) Mean and SEM of CB1 blot densities of each age group (n = 8 hemispheres each from 4 animals, one-way factorial ANOVA, p<0.05, *post hoc* Tukey’s test, *: p<0.05). The blot densities were normalized to the mean density of P10. (C) CB1 immunostaining of the binocular region of V1 at postnatal ages indicated on top. Scale, 100 µm. (D) Layer distribution of CB1 immunoreactivity in the binocular region of V1 at different postnatal ages. Mean and SEM of CB1 signal intensity in each layer represented as the proportion to the all-layer intensity (n = 4 animals, one-way factorial ANOVA, p<0.05; layer II/III, p>0.05; layers IV, V, and VI, *post hoc* Tukey’s test, *: p<0.05, **: p<0.01).

To address the possible role of CB1 in the developmental plasticity of V1, we explored the developmental regulation of CB1 in V1. The relative amount of CB1 protein in V1 gradually increased during development from P10 to P100 (Fig. 3A, B). The relative amount of CB1 at P100 was significantly higher than that at P20 (Fig. 3B). In the mice from P20 to P100, intense CB1 immunoreactivity was mainly observed in layers II/III and VI, while intense immunoreactivity was observed in layers I and VI in P10 animals (Fig. 3C). In layer II/III, the CB1 immunoreactivity between P30 and P50 was significantly higher than that of P10 (Fig. 3D).

### Effect of Dark Rearing on CB1 Expression

**Figure 4:**
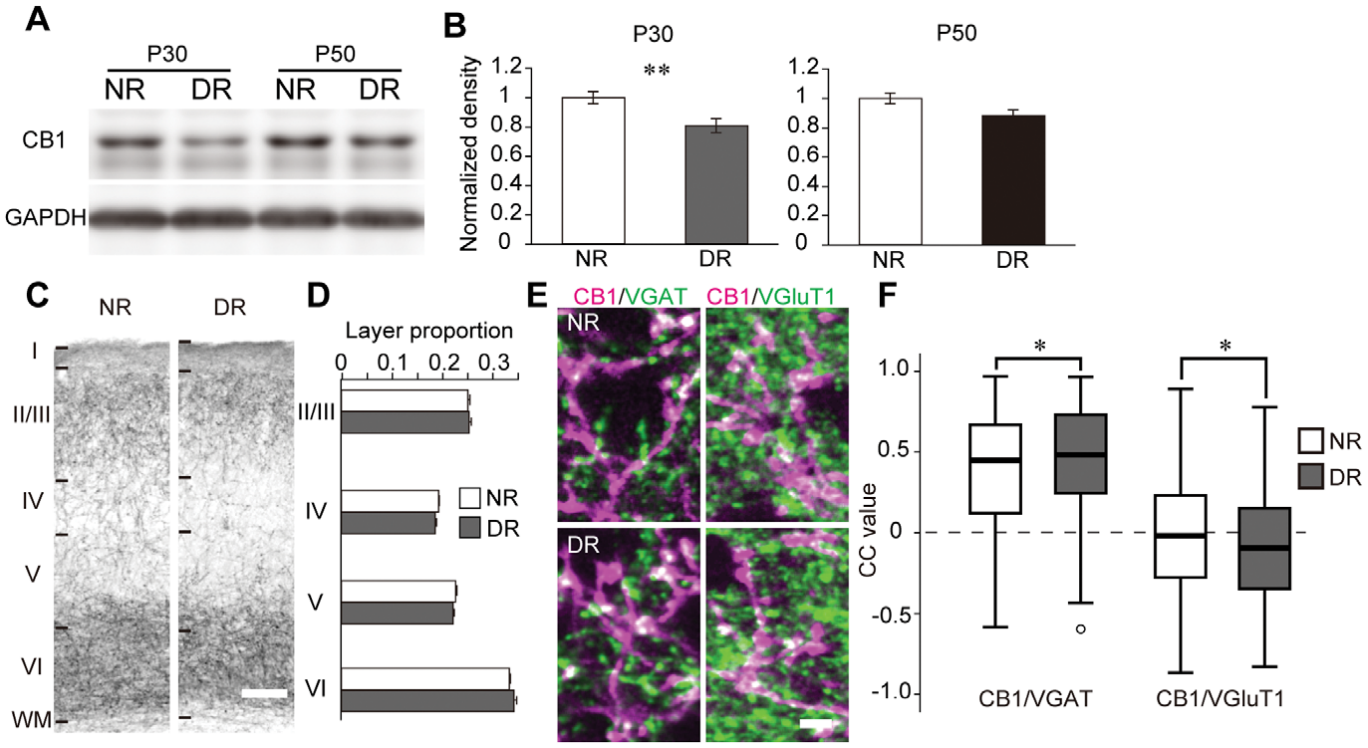
Effects of dark rearing on CB1 expression. (A) Representative western blots of CB1 and GAPDH in V1. The blots of normal light/dark condition-reared (NR) and dark-reared (DR) mice at P30 and P50 are shown. (B) Mean and SEM of the blot density of CB1 (P30: n = 16 (NR) and 21 (DR) animals, P50: n = 5 (NR) and 5 (DR) animals; unpaired t-test, **: p<0.01). (C) Layer distribution of CB1 immunoreactivity in V1. Photographs represent immunostained sections of NR and DR animals at P30. Layer boundaries were determined in neighboring Nissl-stained sections. Scale, 100 µm. (D) CB1 immunoreactivity in individual layers of NR and DR animals at P30. Intensities in each layer are represented as the proportion to the all-layer intensities (two-way ANOVA, p>0.05). (E) Double immunofluorescent staining of CB1 (magenta) and VGAT, VGluT1 in the deep layer of V1 of NR (upper) and DR (lower) animals at P30. Scale, 3 µm. (F) Box and whisker plots showing the CC values of CB1 and VGAT, VGluT1 in the deep layer of NR and DR animals at P30 (n = 3 animals each; NR animals: n = 531 ROIs (CB1/VGAT), 244 ROIs (CB1/VGluT1), DR animals: n = 594 ROIs (CB1/VGAT), 343 ROIs (CB1/VGluT1), Mann-Whitney U test, *: p<0.05).

To explore the effect of visual inputs on the developmental regulation of CB1 expression, we examined CB1 expression in mice that were dark reared from birth to P30 or P50. The mice reared in the dark from birth to P30 had a lesser quantity of CB1 protein than the normal mice reared under normal light/dark conditions. However, the mice that were dark reared until P50 had similar amounts of CB1 protein as the normal mice (Fig. 4A, B). In P30 animals, the pattern of layer distribution of CB1 was similar between the dark-reared and normal groups (Fig. 4C, D). To determine the effect of dark rearing on the synaptic localization of CB1, we compared the colocalization of CB1 and VGAT, VGluT1, or VGluT2 between the dark-reared and normal mice at P30. In the deep layer, the CC value of CB1 and VGAT was significantly higher in the dark-reared mice than that in the normal mice. In contrast, the CC value of CB1 and VGluT1 in the dark-reared mice was significantly lower than that of the normal mice (Fig. 4E, F). In the upper and middle layers, dark rearing did not affect the CC value of CB1 and VGluTs or VGAT.

### Effect of Monocular Deprivation on CB1 Expression

**Figure 5:**
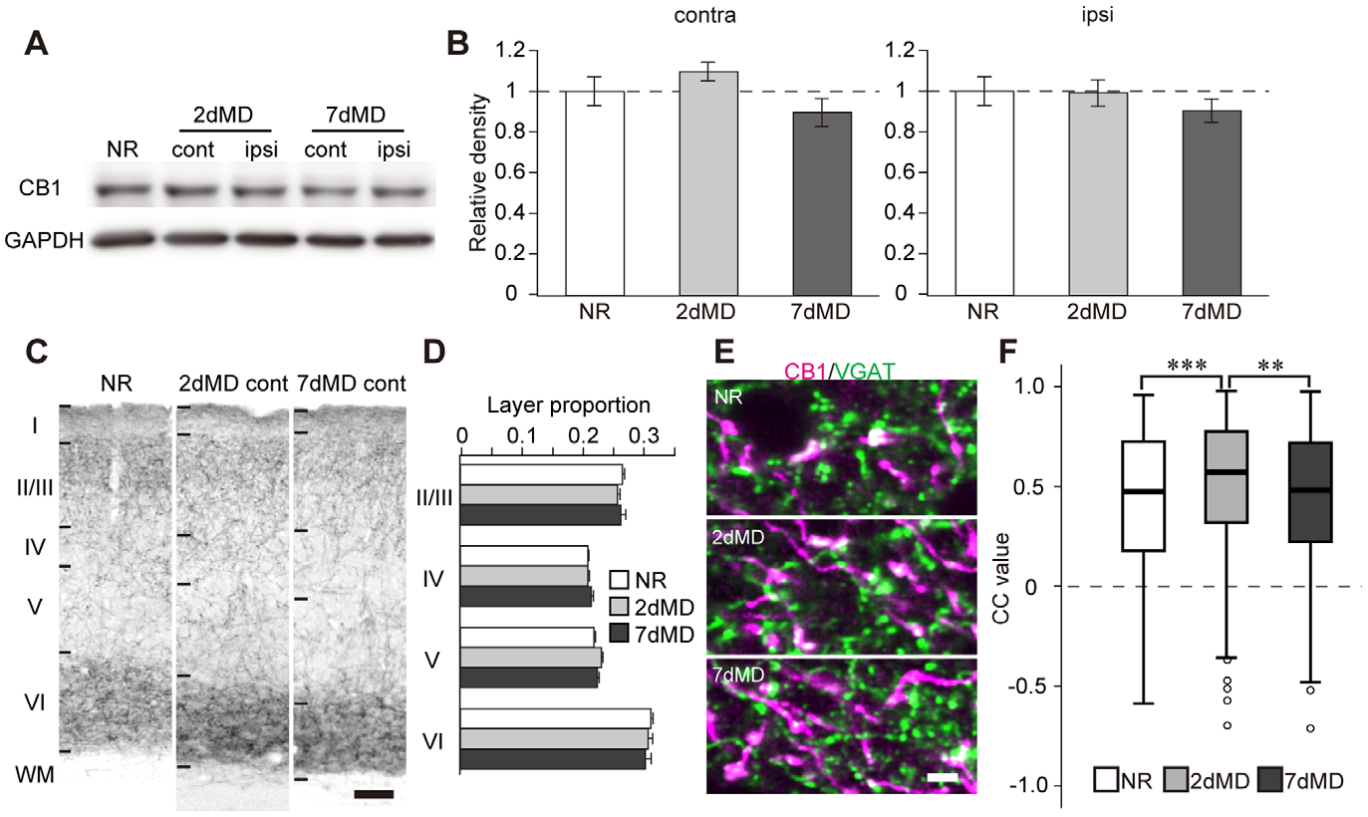
Effects of monocular deprivation on CB1 expression. (A) Representative western blots of CB1 and GAPDH in V1∶2 dMD and 7 dMD indicate monocular deprivation for two days and seven days, respectively. The blots of V1, which is contralateral (cont) or ipsilateral (ipsi) to the deprived eye, are represented with that of the normal animal (NR). (B) Mean and SEM of the blot density of CB1 in MD animals normalized to the mean of normal animals (n = 10 animals each, one-way factorial ANOVA, p>0.05). (C) Representative photographs of CB1 immunoreactivity in V1 of normal and MD animals. Scale, 100 µm. (D) Layer proportion of CB1 immunoreactivity was not significantly different among animal groups (two-way ANOVA, p>0.05). (E) Double immunofluorescent staining of CB1 (magenta) and VGAT (green) in the deep layer of V1 of normal and MD animals. The images of MD animals were obtained in the hemisphere contralateral to the deprived eye. Scale, 3 µm. (F) The CC values of CB1/VGAT in the deep layer of V1, which is contralateral to the deprived eye (n = 3 animals each; n = 386 ROIs (NR), 380 ROIs (2 dMD), 389 ROIs (7 dMD), Bonferroni-corrected Mann-Whitney U-test, **: p<0.0033, ***: p<0.00033).

We examined the effect of monocular deprivation (MD) on CB1 expression and its time course in mice during the critical period of ocular dominance plasticity. MD for 2 days or 7 days did not affect the expression and the layer distribution of CB1 immunoreactivity (Fig. 5A–D). However, in the deep layer of V1, which is contralateral to the deprived eye, the CC value of CB1 and VGAT in 2-day MD mice was significantly higher than that of the normal mice (Fig. 5E, F). On the other hand, the CC value of CB1 and VGluTs did not change significantly following 2 days or 7 days of MD. In the upper and middle layers, MD did not affect the CC value of CB1 and VGluTs or VGAT.

## Discussion

In this study, we examined the postnatal development of protein expression, layer distribution, and synaptic localization of CB1 in the mouse V1, along with the effect of visual experience on these factors. We found that (i) intense CB1 immunoreactivity is mainly observed in layers II/III and VI and localizes at the VGAT-positive inhibitory nerve terminals; (ii) CB1 protein expression increases across development from P10 to P100, and intense CB1 immunoreactivity in layers II/III and VI is observed at P20 and thereafter; (iii) dark rearing from birth to P30 decreases CB1 protein expression in V1 and alters the colocalization of CB1 and VGAT or VGluT1 in the deep layer, although the layer distribution of CB1 remains intact. However, dark rearing until P50 does not affect the expression and distribution of CB1. We also found that (iv) MD for 2 days during the critical period of ODP increases the localization of CB1 at VGAT-positive nerve terminals in the deep layer, while the protein expression and the layer distribution of CB1 are not affected. MD for 7 days does not exert a noticeable effect on the expression and localization of CB1.

### Subcellular Localization of CB1

In the hippocampus and primary somatosensory cortex, CB1 mainly localizes at the cholecystokinin (CCK)-positive inhibitory neurons but not at the parvalbumin (PV)-positive neurons [23], [24], although a recent work has revealed that unitary IPSCs derived from PV neurons are affected by CB1 agonists at high concentrations [17]. In contrast, a high-titer antibody against CB1 detects immunoreactivity at the excitatory nerve terminals in the hippocampus and the cerebellum [25]. In the rat sensorimotor cortex, single cell RT-PCR detects mRNA of CB1 in pyramidal neurons [26]. In addition, electrophysiological studies reported that both excitatory and inhibitory LTD of synaptic transmission require CB1 activity [14]–[18]. These findings suggest that functional CB1 receptors are expressed in both excitatory and inhibitory neurons, although the expression level is higher in inhibitory neurons. In accordance with the previous reports, we found that the colocalization of CB1 and VGAT is significantly higher than that of CB1 and VGluTs in the V1 of P30 mice. Considering that the modulation of PV neuron-derived IPSCs by CB1 agonists diminishes in the V1 at 5 weeks of age [17], CB1 may mainly localize at CCK-positive inhibitory nerve terminals in the mouse V1 at P30.

### Developmental Regulation of CB1

In the binocular region of V1, intense CB1 immunoreactivity in layers II/III and VI was observed at P20 and maintained thereafter to P100. A previous report showed that a CB1 antagonist inhibits the ODP in layer II/III of V1 in mice at P26–31 [13]. In addition, CB1-mediated LTD in layer II/III was reported in juvenile mice [15]–[18]. Our results are consistent with the previous reports because intense CB1 immunoreactivity in layer II/III already exists at the age at which CB1-mediated developmental plasticity takes place. Because P20 is just before the beginning of the critical period of the ODP in mice [2], [27], CB1 expression may contribute to the beginning of the critical period by enabling synaptic plasticity in layer II/III of V1. Although the appearance of CB1 in layer II/III coincides with the beginning of the critical period in V1, the expression and immunoreactivity of CB1 were maintained long after the end of it, until P100. Thus, the closure of the critical period should be regulated by other molecular mechanisms, such as extracellular matrix- or myelin-related molecules [28], [29].

Intense CB1 immunoreactivity in layers II/III and VI is also observed in the primary somatosensory cortex (S1) [20], [24]. In S1, however, the specific laminar pattern of CB1 appears earlier than V1, between P6 and P20 [20]. This difference may underlie the earlier onset of experience-dependent plasticity in S1 than in V1 [2], [30], [31].

Considering the intense immunoreactivity of CB1 after the closure of the critical period, CB1 may play a role in visual processing in the adult V1 by modulating synaptic interactions as observed in the LGN [32]. Because intense CB1 immunoreactivity is observed in layer VI of the adult V1, CB1 may contribute to the visual information processing in the deep layer, such as gain control [33].

### Visual Inputs Contribute to the Developmental Regulation of CB1

Dark rearing from birth disturbs the normal development of visual function, delays the critical period of ODP [4], [34], and alters the expression of various molecules in V1 [6], [7], [9]. In the present experiments, dark rearing from birth to P30 decreased the expression of CB1 protein in V1, though the layer distribution of CB1 was not affected. This result suggests that CB1 expression in layers II/III and VI can proceed in the absence of visual inputs, but the amount of expression is reduced by dark rearing. In the mice reared in the dark from birth to P50, however, the expression level of CB1 was comparable to that of the normal animals. Therefore, visual inputs might play a promoting role in the development of CB1 expression.

We have shown that the colocalization of CB1 and VGAT increases and that of CB1 and VGluT1 decreases, in the deep layer of V1 after dark rearing until P30. This result indicates that the dark-reared mice have more CB1-positive inhibitory nerve terminals and less CB1-positive excitatory nerve terminals than normal mice. Because CB1 negatively regulates neurotransmission, the excitability of the neural circuitry may be augmented in the deep layer of dark-reared mice.

### Monocular Deprivation Affects the Synaptic Localization of CB1 in the Deep Layer

Ocular dominance plasticity is suggested to involve the eCB signal pathway, as a CB1 antagonist was shown to suppress ODP in layer II/III [13]. Because MD first induces a depression of deprived eye responses, which is followed by a potentiation of open eye responses [35], we examined the effect of MD for 2 days and 7 days on CB1 expression. MD of either duration did not influence the amount or the layer distribution of CB1. Therefore, the ODP in layer II/III would require CB1 activity, but not the modification of CB1 expression. As to synaptic localization, the colocalization of CB1 and VGAT transiently increased following 2 days of MD in the deep layer of V1. The transient increase in the colocalization of CB1 and VGAT, together with the similar modification observed in the dark-reared mice at P30, suggests that CB1 expression in the deep layer of V1 is affected by the quantity of visual inputs.

## References

[1] Wiesel TN, Hubel DH (1963) Single-cell responses in striate cortex of kittens deprived of vision in one eye. J Neurophysiol 26: 1003–1017. 14084161 10.1152/jn.1963.26.6.1003

[2] Gordon JA, Stryker MP (1996) Experience-dependent plasticity of binocular responses in the primary visual cortex of the mouse. J Neurosci 16: 3274–3286. 8627365 10.1523/JNEUROSCI.16-10-03274.1996 PMC6579137

[3] Olson CR, Freeman RD (1980) Profile of the sensitive period for monocular deprivation in kittens. Exp Brain Res 39: 17–21. 7379883 10.1007/BF00237065

[4] Mower GD (1991) The effect of dark rearing on the time course of the critical period in cat visual cortex. Brain Res Dev Brain Res 58: 151–158. 2029762 10.1016/0165-3806(91)90001-y

[5] Hensch TK (2005) Critical period plasticity in local cortical circuits. Nat Rev Neurosci 6: 877–888. 16261181 10.1038/nrn1787

[6] Majdan M, Shatz CJ (2006) Effects of visual experience on activity-dependent gene regulation in cortex. Nat Neurosci 9: 650–659. 16582906 10.1038/nn1674

[7] Tropea D, Kreiman G, Lyckman A, Mukherjee S, Yu H, et al (2006) Gene expression changes and molecular pathways mediating activity-dependent plasticity in visual cortex. Nat Neurosci 9: 660–668. 16633343 10.1038/nn1689

[8] Lyckman AW, Horng S, Leamey CA, Tropea D, Watakabe A, et al (2008) Gene expression patterns in visual cortex during the critical period: Synaptic stabilization and reversal by visual deprivation. Proc Natl Acad Sci U S A 105: 9409–9414. 18606990 10.1073/pnas.0710172105 PMC2453704

[9] Dahlhaus M, Li KW, van der Schors RC, Saiepour MH, van Nierop P, et al.. (2011) The synaptic proteome during development and plasticity of the mouse visual cortex. Mol Cell Proteomics 10: M110 005413. 10.1074/mcp.M110.005413 PMC3098591 21398567

[10] Chevaleyre V, Takahashi KA, Castillo PE (2006) Endocannabinoid-mediated synaptic plasticity in the CNS. Annu Rev Neurosci 29: 37–76. 16776579 10.1146/annurev.neuro.29.051605.112834

[11] Kano M, Ohno-Shosaku T, Hashimotodani Y, Uchigashima M, Watanabe M (2009) Endocannabinoid-mediated control of synaptic transmission. Physiol Rev 89: 309–380. 19126760 10.1152/physrev.00019.2008

[12] Tanimura A, Yamazaki M, Hashimotodani Y, Uchigashima M, Kawata S, et al (2010) The endocannabinoid 2-arachidonoylglycerol produced by diacylglycerol lipase α mediates retrograde suppression of synaptic transmission. Neuron 65: 320–327. 20159446 10.1016/j.neuron.2010.01.021

[13] Liu CH, Heynen AJ, Shuler MG, Bear MF (2008) Cannabinoid receptor blockade reveals parallel plasticity mechanisms in different layers of mouse visual cortex. Neuron 58: 340–345. 18466745 10.1016/j.neuron.2008.02.020 PMC12999183

[14] Sjöström PJ, Turrigiano GG, Nelson SB (2003) Neocortical LTD via coincident activation of presynaptic NMDA and cannabinoid receptors. Neuron 39: 641–654. 12925278 10.1016/s0896-6273(03)00476-8

[15] Crozier RA, Wang Y, Liu CH, Bear MF (2007) Deprivation-induced synaptic depression by distinct mechanisms in different layers of mouse visual cortex. Proc Natl Acad Sci U S A 104: 1383–1388. 17227847 10.1073/pnas.0609596104 PMC1783104

[16] Huang Y, Yasuda H, Sarihi A, Tsumoto T (2008) Roles of endocannabinoids in heterosynaptic long-term depression of excitatory synaptic transmission in visual cortex of young mice. J Neurosci 28: 7074–7083. 18614676 10.1523/JNEUROSCI.0899-08.2008 PMC6670480

[17] Jiang B, Huang S, de Pasquale R, Millman D, Song L, et al (2010) The maturation of GABAergic transmission in visual cortex requires endocannabinoid-mediated LTD of inhibitory inputs during a critical period. Neuron 66: 248–259. 20435001 10.1016/j.neuron.2010.03.021 PMC2897012

[18] Jiang B, Sohya K, Sarihi A, Yanagawa Y, Tsumoto T (2010) Laminar-specific maturation of GABAergic transmission and susceptibility to visual deprivation are related to endocannabinoid sensitivity in mouse visual cortex. J Neurosci 30: 14261–14272. 20962247 10.1523/JNEUROSCI.2979-10.2010 PMC6634750

[19] Chaves GP, Nogueira TCA, Britto LRG, Bordin S, Torrão AS (2008) Retinal removal up-regulates cannabinoid CB1 receptors in the chick optic tectum. J Neurosci Res 86: 1626–1634. 18189324 10.1002/jnr.21613

[20] Deshmukh S, Onozuka K, Bender KJ, Bender VA, Lutz B, et al (2007) Postnatal development of cannabinoid receptor type 1 expression in rodent somatosensory cortex. Neuroscience 145: 279–287. 17210229 10.1016/j.neuroscience.2006.11.033 PMC1850104

[21] Fukudome Y, Ohno-Shosaku T, Matsui M, Omori Y, Fukaya M, et al (2004) Two distinct classes of muscarinic action on hippocampal inhibitory synapses: M2-mediated direct suppression and M1/M3-mediated indirect suppression through endocannabinoid signalling. Eur J Neurosci 19: 2682–2692. 15147302 10.1111/j.0953-816X.2004.03384.x

[22] Uchigashima M, Narushima M, Fukaya M, Katona I, Kano M, et al (2007) Subcellular arrangement of molecules for 2-arachidonoyl-glycerol-mediated retrograde signaling and its physiological contribution to synaptic modulation in the striatum. J Neurosci 27: 3663–3676. 17409230 10.1523/JNEUROSCI.0448-07.2007 PMC6672418

[23] Katona I, Sperlagh B, Sik A, Kafalvi A, Vizi ES, et al (1999) Presynaptically located CB1 cannabinoid receptors regulate GABA release from axon terminals of specific hippocampal interneurons. J Neurosci 19: 4544–4558. 10341254 10.1523/JNEUROSCI.19-11-04544.1999 PMC6782612

[24] Bodor AL, Katona I, Nyiri G, Mackie K, Ledent C, et al (2005) Endocannabinoid signaling in rat somatosensory cortex: laminar differences and involvement of specific interneuron types. J Neurosci 25: 6845–6856. 16033894 10.1523/JNEUROSCI.0442-05.2005 PMC6725346

[25] Kawamura Y, Fukaya M, Maejima T, Yoshida T, Miura E, et al (2006) The CB1 cannabinoid receptor is the major cannabinoid receptor at excitatory presynaptic sites in the hippocampus and cerebellum. J Neurosci 26: 2991–3001. 16540577 10.1523/JNEUROSCI.4872-05.2006 PMC6673964

[26] Hill EL, Gallopin T, Ferezou I, Cauli B, Rossier J, et al (2007) Functional CB1 receptors are broadly expressed in neocortical GABAergic and glutamatergic neurons. J Neurophysiol 97: 2580–2589. 17267760 10.1152/jn.00603.2006

[27] Fagiolini M, Hensch TK (2000) Inhibitory threshold for critical-period activation in primary visual cortex. Nature 404: 183–186. 10724170 10.1038/35004582

[28] Pizzorusso T, Medini P, Berardi N, Chierzi S, Fawcett JW, et al (2002) Reactivation of ocular dominance plasticity in the adult visual cortex. Science 298: 1248–1251. 12424383 10.1126/science.1072699

[29] McGee AW, Yang Y, Fischer QS, Daw NW, Strittmatter SM (2005) Experience-driven plasticity of visual cortex limited by myelin and Nogo receptor. Science 309: 2222–2226. 16195464 10.1126/science.1114362 PMC2856689

[30] Fox K (1992) A critical period for experience-dependent synaptic plasticity in rat barrel cortex. J Neurosci 12: 1826–1838. 1578273 10.1523/JNEUROSCI.12-05-01826.1992 PMC6575898

[31] Stern EA, Maravall M, Svoboda K (2001) Rapid development and plasticity of layer 2/3 maps in rat barrel cortex in vivo. Neuron 31: 305–315. 11502260 10.1016/s0896-6273(01)00360-9

[32] Dasilva MA, Grieve KL, Cudeiro J, Rivadulla C (2011) Endocannabinoid CB1 receptors modulate visual output from the thalamus. Psychopharmacology 219: 835–845. 21773721 10.1007/s00213-011-2412-3

[33] Olsen SR, Bortone DS, Adesnik H, Scanziani M (2012) Gain control by layer six in cortical circuits of vision. Nature 483: 47–52. 22367547 10.1038/nature10835 PMC3636977

[34] Gianfranceschi L, Siciliano R, Walls J, Morales B, Kirkwood A, et al (2003) Visual cortex is rescued from the effects of dark rearing by overexpression of BDNF. Proc Natl Acad Sci U S A 100: 12486–12491. 14514885 10.1073/pnas.1934836100 PMC218784

[35] Frenkel MY, Bear MF (2004) How monocular deprivation shifts ocular dominance in visual cortex of young mice. Neuron 44: 917–923. 15603735 10.1016/j.neuron.2004.12.003

